# Providing quality care for people with CDKL5 deficiency disorder: A European expert panel opinion on the patient journey

**DOI:** 10.1002/epi4.12914

**Published:** 2024-03-07

**Authors:** Sam Amin, Rikke S. Møller, Angel Aledo‐Serrano, Alexis Arzimanoglou, Patrick Bager, Sergiusz Jóźwiak, Gerhard Josef Kluger, Sandra López‐Cabeza, Rima Nabbout, Carol‐Anne Partridge, Susanne Schubert‐Bast, Nicola Specchio, Reetta Kälviäinen

**Affiliations:** ^1^ University Hospitals Bristol Bristol UK; ^2^ The Danish Epilepsy Centre, Filadelfia Dianalund Denmark; ^3^ Department of Regional Health Research, Faculty of Health Sciences University of Southern Denmark Odense Denmark; ^4^ Vithas Madrid La Milagrosa University Hospital Vithas Hospital Group Madrid Spain; ^5^ San Juan de Dios Children's Hospital Barcelona Spain; ^6^ CDKL5 Deutschland e.V. Mainz Germany; ^7^ The Children's Memorial Health Institute Warsaw Poland; ^8^ Epilepsy Center for Children and Adolescents Vogtareuth Germany; ^9^ Paracelsus Medical University Salzburg Salzburg Austria; ^10^ Asociación de Afectados CDKL5 Madrid Spain; ^11^ Necker‐Enfants Malades Hospital Université Paris Cité, Imagine Institute Paris France; ^12^ CDKL5 UK Somerset UK; ^13^ Center of Neurology and Neurosurgery Epilepsy Center Frankfurt Rhine‐Main Goethe‐University and University Hospital Frankfurt Frankfurt am Main Germany; ^14^ LOEWE Center for Personalized and Translational Epilepsy Research (CePTER) Goethe‐University Frankfurt am Main Germany; ^15^ University Children's Hospital Goethe‐University and University Hospital Frankfurt Frankfurt am Main Germany; ^16^ Bambino Gesù Children's Hospital IRCCS Rome Italy; ^17^ University of Eastern Finland and Epilepsy Center Kuopio University Hospital Kuopio Finland

**Keywords:** cyclin‐dependent kinase‐like 5, developmental and epileptic encephalopathy, diagnosis, multidisciplinary care

## Abstract

Cyclin‐dependent kinase‐like 5 (CDKL5) deficiency disorder (CDD) is a developmental and epileptic encephalopathy caused by variants in the *CDKL5* gene. The disorder is characterized by intractable early‐onset seizures, severe neurodevelopmental delay, hypotonia, motor disabilities, cerebral (cortical) visual impairment and microcephaly. With no disease‐modifying therapies available for CDD, treatment is symptomatic with an initial focus on seizure control. Another unmet need in the management of people with CDD is the lack of evidence to aid standardized care and guideline development. To address this gap, experts in CDD and representatives from patient advocacy groups from Denmark, Finland, France, Germany, Italy, Poland, Spain, and the United Kingdom convened to form an Expert Working Group. The aim was to provide an expert opinion consensus on how to ensure quality care in routine clinical practice within the European setting, including in settings with limited experience or resources for multidisciplinary care of CDD and other developmental and epileptic encephalopathies. By means of one‐to‐one interviews around the current treatment landscape in CDD, insights from the Expert Working Group were collated and developed into a Europe‐specific patient journey for individuals with CDD, which was later validated by the group. Further discussions followed to gain consensus of opinions on challenges and potential solutions for achieving quality care in this setting. The panel recognized the benefit of early genetic testing, a holistic personalized approach to seizure control (taking into consideration various factors such as concomitant medications and comorbidities), and age‐ and comorbidity‐dependent multidisciplinary care for optimizing patient outcomes and quality of life. However, their insights and experiences also highlighted much disparity in management approaches and resources across different European countries. Development of standardized European recommendations is required to align realistic diagnostic criteria, treatment goals, and management approaches that can be adapted for different settings.

**Plain language summary:**

Cyclin‐dependent kinase‐like 5 (CDKL5) deficiency disorder (CDD) is a rare condition caused by a genetic mutation with a broad range of symptoms apparent from early childhood, including epileptic seizures that do not respond to medication and severe delays in development. Due to the lack of guidance on managing CDD, international experts and patient advocates discussed best practices in the care of people with CDD in Europe. The panel agreed that early testing, a personalized approach to managing seizures, and access to care from different disciplines are beneficial. Development of guidelines to ensure that care is standardized would also be valuable.

**Key points**

- Diagnosis by key clinical features and genetic testing is beneficial for early CDD diagnosis, managing seizures, and optimizing patient outcomes.
- Several factors (e.g., concomitant medications and comorbidities) should be considered when deciding how to manage seizures.
- Realistic expectations should be communicated to patients/family/caregivers about the difficulties in achieving seizure freedom.
- Multidisciplinary care should be individualized based on patients' comorbidities and age—although such care is often limited by time and available resources.
- Many challenges (and solutions) associated with CDD management approaches may be applicable to other developmental and epileptic encephalopathies.

## 1 INTRODUCTION

Cyclin‐dependent kinase‐like 5 (CDKL5) deficiency disorder (CDD)^1^ falls under the developmental and epileptic encephalopathy (DEE)^2^ group of diseases. DEEs are characterized by impaired neurologic development; a consequence of the underlying etiology and epileptic activity.^3^ Owing to some overlapping (but distinct) clinical features, CDD was initially identified as an early‐onset variant of Rett syndrome,^1^, ^4^ but research demonstrating distinguishable symptoms and genetic cause led to CDD being considered an independent clinical condition. CDD is an X‐linked disorder affecting approximately 1 in 40 000–60 000 live births,^5^ caused by pathogenic variants in the *CDKL5* gene,^6^ which encodes a serine/threonine kinase essential for normal brain development and function.^5^, ^7^ CDD presents in more females than males (at a ratio of 4:1),^1^ although male patients often display more severe symptoms than female patients.^5^, ^8^, ^9^ CDD covers a broad range of clinical features including early‐onset refractory epilepsy; hypotonia; developmental, motor, and intellectual disabilities; cerebral (cortical) visual impairment (CVI)^5^; and microcephaly.^10^ Of these symptoms, epileptic seizures are usually the first to occur, with a median age at onset of 6 weeks (onset by 3 months in 90% of cases).^5^, ^11^, ^12^

There are currently no disease‐modifying therapies available for CDD. Rather, patients are treated using a multidisciplinary care approach that focuses on prompt seizure management, as well as addressing associated comorbidities.^5^, ^13^ With approximately 70% of children with CDD experiencing daily seizures, the most common treatment is the use of antiseizure medications (ASMs) to reduce the frequency and severity of seizures.^12^ Various ASMs have been found to be effective in the short‐term treatment of CDD‐related seizures, although efficacy wanes over time. According to the International CDKL5 Disorder Database, the ASMs most frequently prescribed for CDD include clobazam, valproate, topiramate, levetiracetam, and vigabatrin.^14^ In a retrospective evaluation of long‐term effectiveness of conventional ASMs, clobazam, felbamate, lamotrigine, vigabatrin, valproate, zonisamide, and steroids demonstrated highest rates of seizure reduction at 3 months.^15^

While there are no approved ASMs for CDD, there are a handful of ASMs that have been approved for specific use across a range of DEEs. For example, cannabidiol is indicated for use in people aged 2 years or over to treat seizures related to tuberous sclerosis complex (TSC), Dravet syndrome (as add‐on to clobazam in the European Union), and Lennox–Gastaut syndrome (as add‐on to clobazam in the European Union).^16^ Although everolimus is not an ASM, it is a mammalian target of rapamycin inhibitor that is indicated for treatment of focal‐onset seizures related to TSC in patients from 2 years of age who have not responded to other treatments.^17^ Stiripentol is indicated for adjunctive use with clobazam and valproate to treat refractory‐generalized tonic–clonic seizures in patients with Dravet syndrome.^18^, ^19^ Fenfluramine is indicated for the treatment of seizures associated with Dravet syndrome in patients aged 2 years or older.^20^ Valproic acid is indicated for the treatment of generalized, focal, or other epilepsies.^21^, ^22^, ^23^, ^24^ Vigabatrin is indicated for treatment in combination with other ASMs of patients with resistant partial epilepsy (with or without secondary generalization) and monotherapy of patients with infantile epileptic spasms syndrome (IESS; formerly West syndrome).^25^ Common non‐pharmacological approaches to treating epilepsy in CDD include ketogenic diet and vagus nerve stimulation, both of which have demonstrated effectiveness regarding seizure control.^1^, ^26^, ^27^ Additional, less well‐known and less well‐studied complementary therapies that may be beneficial as a complement to ASMs and other non‐pharmacological interventions in the management of epilepsies include lifestyle interventions, nutritional supplements, music therapy, and herbal remedies.^28^, ^29^, ^30^, ^31^, ^32^

Non‐pharmacological treatments that are part of the multidisciplinary care surrounding the non‐seizure symptoms of CDD include speech therapy, physiotherapy, psychosocial support, and occupational therapy.^5^, ^13^ There is also evidence to suggest that early use of neuro‐visual rehabilitation through stimulation of eye movements can enhance development based on neuronal plasticity.^33^

The International League Against Epilepsy (ILAE) developed a modified classification and framework for seizures in the neonatal period,^34^ in line with 2017 ILAE classifications,^35^ as neonatal seizures may not fit easily into classification schemes for seizures and epilepsies primarily developed for older children and adults. It further proposed classification and definition of epilepsy syndromes with onset in neonates and infants, including CDD.^3^, ^34^ Despite this availability of guidance on defining CDD, due to a paucity of evidence, there is a lack of standardized care and clinical management guidelines, as confirmed by a comprehensive literature review performed by Amin S et al and published as part of an international consensus recommendation from international healthcare professionals (HCPs) for the clinical management of CDD.^36^

The international consensus recommendations^36^ will help guide and improve medical care of patients with CDD. The current project set out to map the patient journey for people with CDD within the European setting. Then, based on this insight, further provide expert opinion on how to ensure quality care in routine clinical practice, with discussion on challenges and potential solutions that can be applied in the care for people with CDD (and potentially other DEEs) across Europe. Ultimately, the aim was to provide evidence‐ and experience‐based recommendations on the diagnosis and care of people with CDD that can also be used outside of clinics specializing in the multidisciplinary care of rare genetic epilepsy syndromes.

## 2 METHODS

**FIGURE 1:**
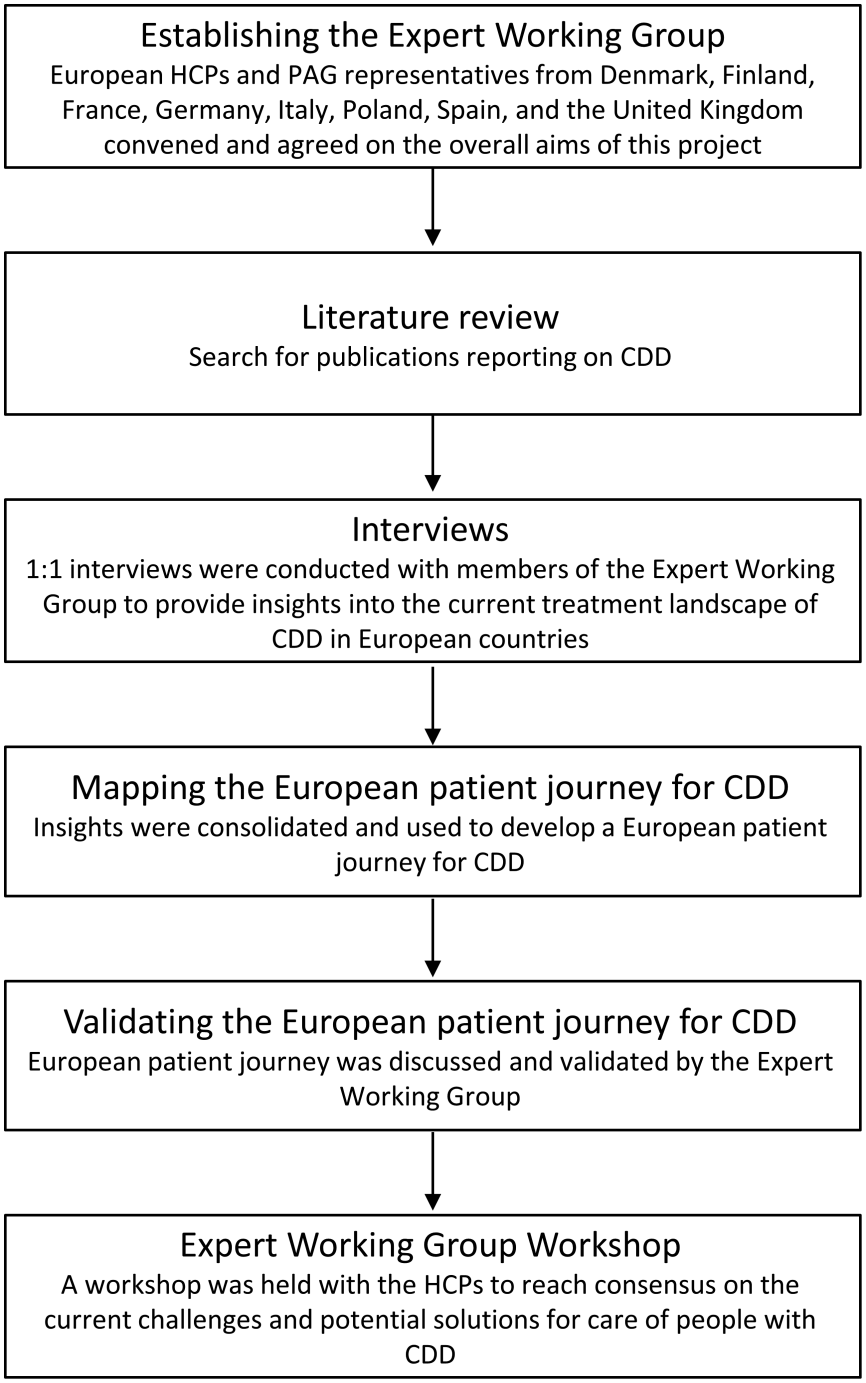
Identification of patient journey and challenges and potential solutions for the care of people with CDD in Europe. CDD, CDKL5 deficiency disorder; HCP, healthcare professional; PAG, patient advocacy group.

Establishing expert recommendations for achieving quality care for individuals with CDD within Europe was endeavored through a multi‐part approach that included mapping the key elements of the current treatment and patient pathways in Europe for CDD, identifying current issues/challenges in care for this population, and recommending potential approaches or solutions to the challenges. The steps involved in this process are outlined in Figure 1.

### 2.1 Establishing the Expert Working Group

A group of three representatives from patient advocacy groups (PAGs) from Germany, Spain, and the United Kingdom, and 10 HCPs from across Europe (Denmark, Finland, France, Germany, Italy, Poland, Spain, and the United Kingdom; most of whom are members of the European Reference Network for Rare and Complex Epilepsies [ERN EpiCARE]), with expertise in CDD management, formed an Expert Working Group. This working group was established to explore current treatment and patient pathways, and to identify research and educational needs and common goals to improve diagnosis, treatment, and care in the setting of CDD.

### 2.2 Literature review

A broad literature review was performed through a search of PubMed for publications on guidelines for the diagnosis and management of CDD published between December 13, 2019, and December 13, 2022. The search results were made available to all co‐authors. A sparsity of evidence to develop guidelines for the management of CDD was detected and—as a further step—this consensus expert opinion piece was advocated to fill the gaps.

### 2.3 One‐to‐one interviews

To reduce the risk of bias in the consensus process, a third‐party agency was commissioned to conduct individual semi‐structured interviews between January and March 2022 with each member of the Expert Working Group. The interviews were conducted with the HCPs on the current management of CDD and patient journey in European countries (key interview topics shown in Table 1) and with the PAG representatives to understand their perspective on the patient journey and treatment of CDD, as well as the role of caregivers in the management of CDD. Insights gathered were consolidated and used to develop a Europe‐specific patient journey for individuals with CDD. In March 2022, the Expert Working Group met to further discuss and validate the European patient journey.

**TABLE 1:** Insights into the current CDD patient journey.

Key discussion points for one‐to‐one interviews with HCPs
Unmet medical needs for patients with CDD
Key HCPs involved in the management of patients with CDD
Identification of challenges for non‐specialist physicians treating patients with CDD, including those involved in genetic testing
Pharmacological therapies used in clinical practice to treat seizures and comorbidities, and the impact of age on the approach to treatment
Collaboration between specialist centers and other HCPs involved in the management of CDD

Abbreviations: CDD, CDKL5 deficiency disorder; HCP, healthcare professional.

### 2.4 Expert Working Group workshop

A workshop with the HCPs of the Expert Working Group was held on October 7, 2022, to identify current challenges and solutions for the establishment of optimal care of people with CDD across care settings in Europe. During this workshop, the groups focused discussions on three main sections of the patient journey identified in the Expert Working Group interviews, namely (1) clinical presentation and diagnosis, (2) management of seizures, and (3) multidisciplinary care.

## 3 RESULTS

### 3.1 European patient journey for people with CDD

The fully developed and validated patient journey focuses on three main elements in the care of people with CDD; presentation and diagnosis, management of seizures, and multidisciplinary care (Figure 2A).

**FIGURE 2A:**
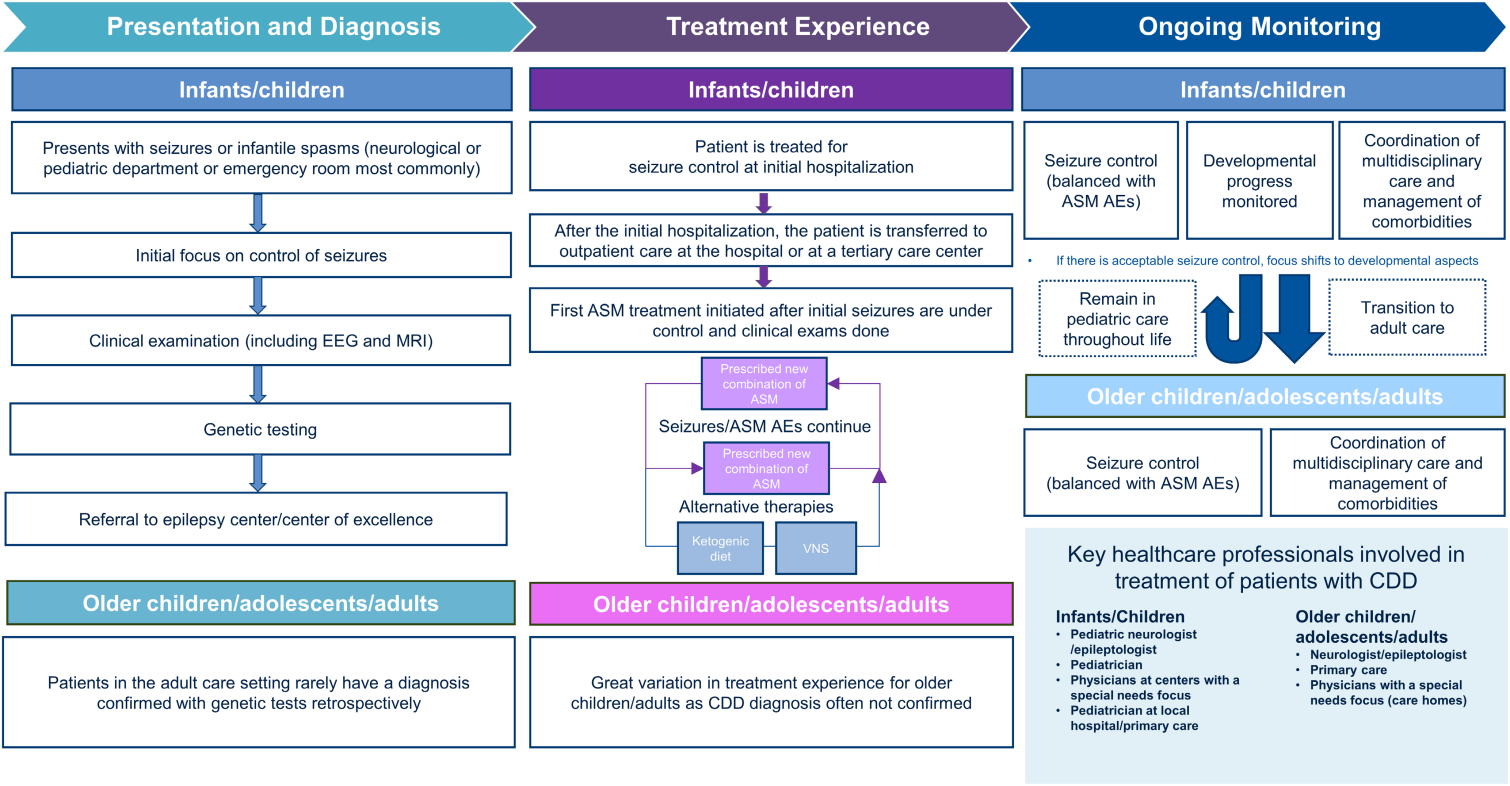
The CDD patient journey in Europe. AE, adverse event; ASM, antiseizure medication; CDD, CDKL5 deficiency disorder; EEG, electroencephalogram; MRI, magnetic resonance imaging; VNS, vagus nerve stimulation.

The Expert Working Group raised several challenges, including the clear practice differences and disparity between countries in various aspects of the European patient journey, including the use, availability, and reimbursement of genetic testing and access to and coordination of multidisciplinary care.

#### 3.1.1 Presentation and diagnosis

Individuals with CDD generally present with seizures, at which point the focus is on seizure control.

Early diagnosis is crucial to patient outcomes in many developmental disorders, including CDD.^37^ Early clinical examinations and testing, such as (video‐)electroencephalogram (EEG) and brain imaging, are components of characterizing the seizures and disorder but may not provide a definite diagnosis.^38^ Therefore, genetic testing should be performed to identify or confirm the specific disorder (especially when there are difficulties distinguishing from similar disorders) (Figure 2B).

**FIGURE 2B:**
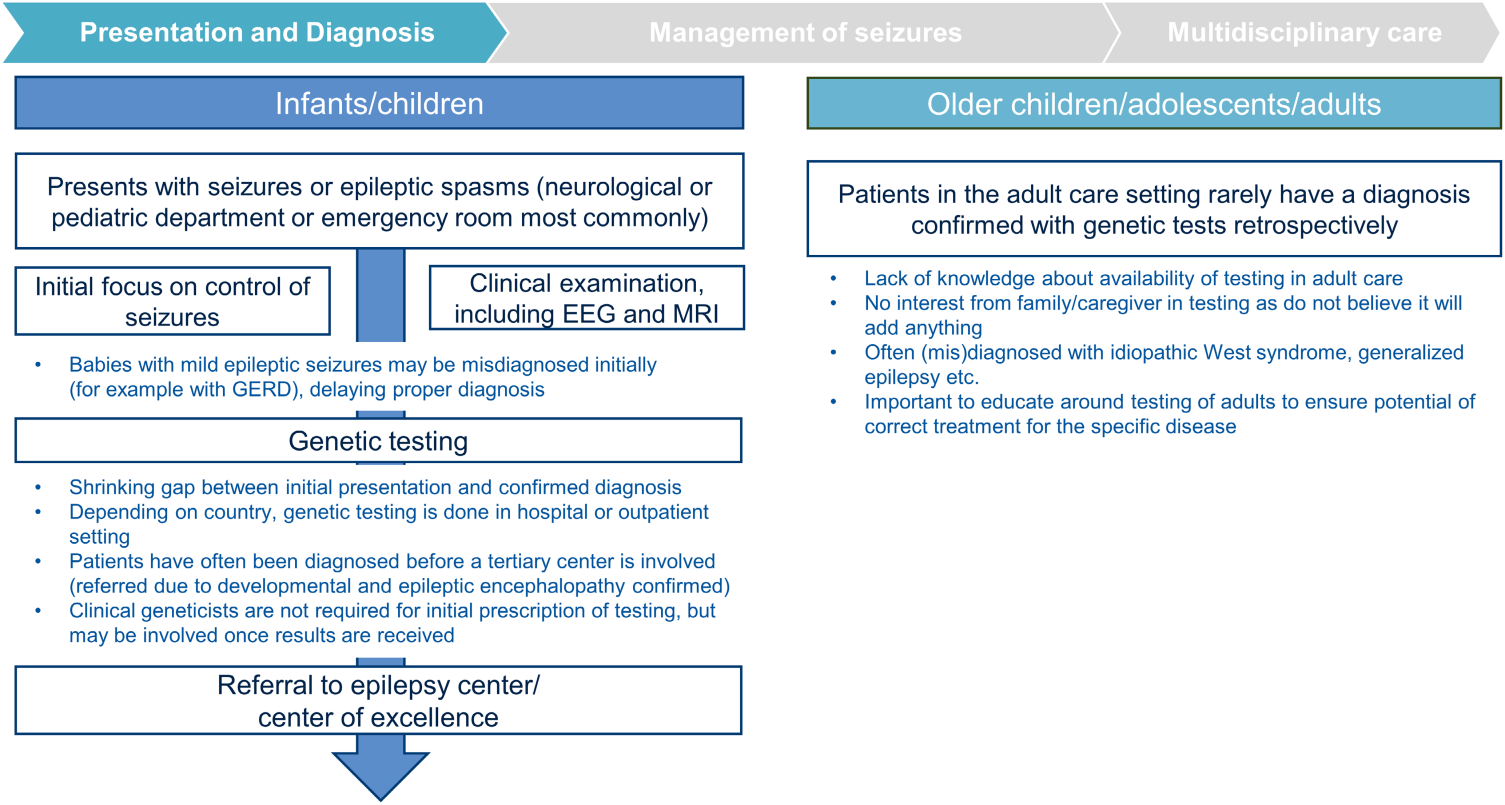
The CDD patient journey in Europe. EEG, electroencephalogram; MRI, magnetic resonance imaging.

#### 3.1.2 Management of seizures

With no disease‐modifying therapies available for the management of CDD, treatment for seizures among individuals with CDD is selected based on the seizure types/patterns at that given period in time, rather than the disorder itself, the tendency for the effectiveness of ASMs to wane over time (the initial phase of seizure‐free periods or significant reduction in seizure frequency after starting an ASM is often termed the “honeymoon effect”),^36^ as well as the effects of polypharmacy and safety profiles of concomitant medications. The severity of refractory epilepsy means that complete seizure freedom is very rarely achieved and often causes patients to cycle through a range of ASMs in seeking to achieve adequate seizure control.^39^ The complexity of management underscores the need for early referral to or consultation of specialized centers, with practitioners knowledgeable in drug management and drug interactions of ASMs in DEEs. Additional non‐pharmacological therapies such as ketogenic diet and vagus nerve stimulation may also be considered part of epilepsy management (Figure 2C).

**FIGURE 2C:**
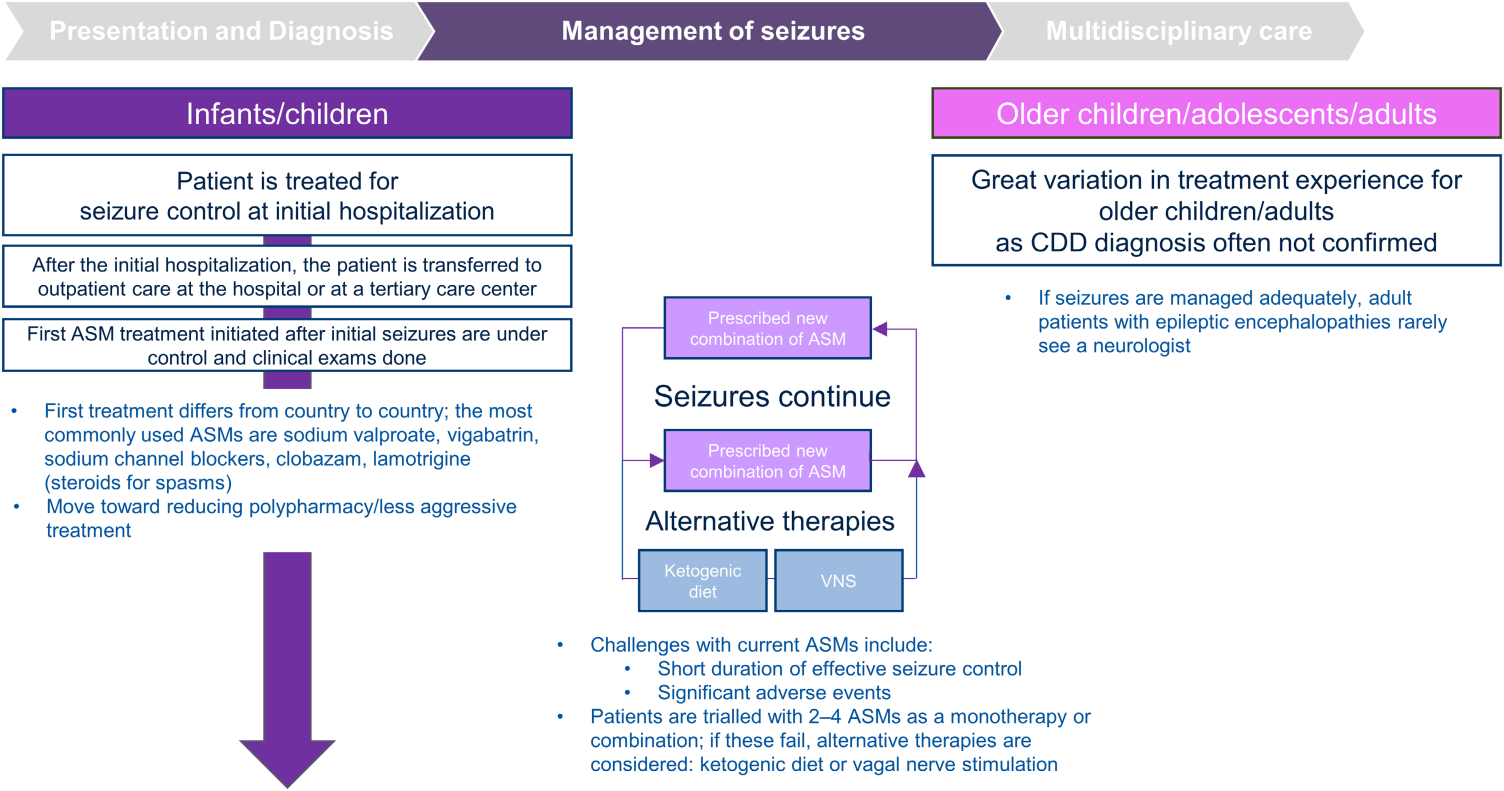
The CDD patient journey in Europe. ASM, antiseizure medication; CDD, CDKL5 deficiency disorder.

#### 3.1.3 Multidisciplinary care

Although seizures are usually the initial focus of treatment, other aspects of the disease have a big impact on quality of life and should be taken into consideration. CDD is complex, and multidisciplinary collaboration and access to specialist centers is essential for long‐term care and support is key (Figure 2D), including a multidisciplinary team with several specialist practitioners (e.g., neurologists, cognitive care specialists, dietitians, gastroenterologists, physiotherapists, and orthopedic specialists).

**FIGURE 2D:**
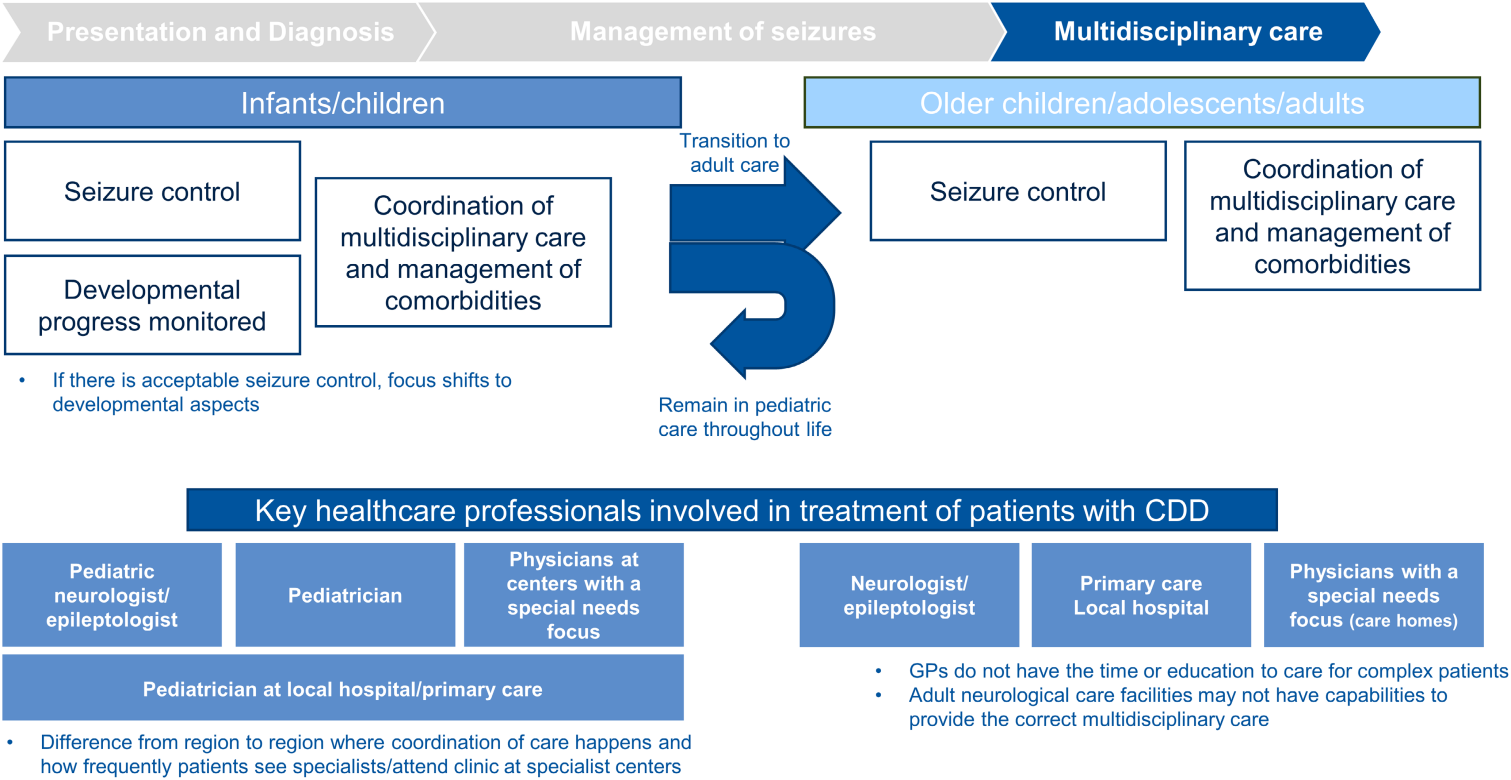
The CDD patient journey in Europe. CDD, CDKL5 deficiency disorder.

Healthcare structures vary across Europe; some healthcare centers are comprised of many different specialists and care teams, whereas others have fewer facilities and need support from relevant working groups or charities. Likewise, patients may transition to adult care in some countries, but in other countries, patients remain in pediatric care throughout their life.

### 3.2 CDD management: current challenges and potential solutions

In the October 2022 workshop for the Expert Working Group, discussions between HCPs highlighted challenges in the management of people with CDD. The group deliberated and offered potential solutions or approaches and directions for the future.

#### 3.2.1 Presentation and diagnosis

##### Clinical features of CDD may be variable

Key clinical features of CDD are age dependent (Table 2). Infants with CDD commonly present in the first few months of life with seizures and pronounced hypotonia; multiple seizure types can occur and they can vary between individuals, but common seizure types include tonic seizures, epileptic spasms and generalized tonic–clonic seizures, and focal seizures.^14^ Infants/young children with CDD experience developmental delays,^4^, ^9^, ^11^, ^40^ refractory seizures,^11^, ^41^, ^42^ severe hypotonia,^4^ CVI,^41^, ^43^, ^44^, ^45^ autistic features,^4^ and hand stereotypies.^4^, ^11^ Both adolescents and adults with CDD frequently experience symptoms such as motor disorders,^4^, ^9^ refractory seizures,^41^ severe hypotonia, autistic features,^4^ and/or hand stereotypies.^4^, ^11^ Severe to profound global delay is present in most cases and affects developmental milestones across age‐groups.

**TABLE 2:** Key clinical features and multidisciplinary treatment goals of CDD according to age.^14^

Age	Key clinical features	Multidisciplinary treatment goals^36^
*Infant*		
>3 months	Median onset of epilepsy 6 weeks^62^ Early focal seizures and/or spasms^40^ EEG can be normal or mildly abnormal^38^ Severe hypotonia^4^ Delay in early developmental milestones^4^, ^9^, ^11^, ^40^	Seizure control/reduced frequency Improve motor function, cognition, behavior, vision, speed, and autonomic function Developmental checks Annual assessments (GI complications, feeding/swallowing, breathing disorders, skin checks, bladder‐related issues, dental checks, and pressure ulcers/skin breakdown) Baseline assessments (speech therapy and cardiac screening/ECG)
3 months–2 years	Onset of seizures by 1 year of age in 90%^11^ Refractory seizures may have honeymoon period^41^, ^42^ May present as IESS (infantile epileptic spasm syndrome)^1^, ^4^ EEG background is progressively abnormal^42^, ^43^ Severe hypotonia^4^ Severe developmental delay and CVI +/− periods of regression^4^, ^41^, ^43^, ^76^ Autistic‐like features^4^ Hand stereotypies^4^, ^11^ Sleep dysfunction^4^ GI dysfunction^11^ Microcephaly^10^	
*Child and adolescent*	Refractory seizures^11^, ^41^ May present as Lennox–Gastaut syndrome^14^ Severe hypotonia^4^ Severe motor delays^4^, ^11^ Severe cognitive impairment^77^ May have experienced regression with or without exacerbation of seizures^11^ Sleep dysfunction^4^ GI dysfunction^11^ Autistic‐like features^4^ Hand stereotypies^4^, ^11^ Simple communication^77^ Microcephaly^10^	Seizure control/reduced frequency Improve motor function, cognition, behavior, vision, speech, and autonomic function Physical therapy Educational support Developmental checks Annual assessments (GI complications, feeding/swallowing, breathing disorders, skin checks, bladder‐related issues, dental checks, and pressure ulcers/skin breakdown) Baseline assessments (speech therapy and cardiac screening/ECG)
*Adult*	Refractory seizures^41^ Severe hypotonia^4^ Severe motor delays^4^ Severe cognitive impairment^77^ May have experienced regression with or without exacerbation of seizures^11^ Sleep dysfunction^4^ GI dysfunction^11^ Autistic‐like features^4^ Hand stereotypies^4^, ^11^ Simple communication^77^ Microcephaly^10^	Seizure control/reduced frequency Improve motor function, cognition, behavior, vision, speed, and autonomic function Physical therapy Occupational therapy Developmental checks Annual assessments (GI complications, feeding/swallowing, breathing disorders, skin checks, bladder‐related issues, dental checks, and pressure ulcers/skin breakdown) Baseline assessments (speech therapy and cardiac screening/ECG)

Abbreviations: CVI, cerebral (cortical) visual impairment; ECG, electrocardiogram; EEG, electroencephalogram; GI, gastrointestinal; IESS, infantile epileptic spasms syndrome.

Although CDD can be suspected based on clinical examination, challenges exist relating to diagnosis based on this only. Despite the defining features of CDD, there may be phenotypic variations between individuals in terms of severity and range of symptoms.^5^, ^14^, ^46^ Owing to the historical association with Rett syndrome, there may be cases of CDD that have been miscategorized as having atypical, *MECP2* mutation negative or early‐onset Rett syndrome. This may be especially true in older individuals or those not affiliated with a center with access to sensitive genetic tests. Additionally, in some cases, CDD may be characterized as IESS, without delving deeper into etiology, based on clinical symptoms.

In CDD and other DEEs, initial seizure types can vary and the type of seizures are not characteristic for the particular disease; over time, seizure types and patterns may change for the individual.^3^

###### Expert opinion

The nosology and definition of epilepsy syndromes in neonates and infants with seizure onset up to 2 years of age, published by the ILAE,^3^, ^34^ was developed to support epilepsy diagnosis. This guidance reports epidemiology, clinical course, seizure types, EEG, neuroimaging, genetics, and differential diagnosis.

Increased awareness of typical as well as rare electroclinical symptoms of CDD would support the important steps of disease recognition, early diagnosis, and appropriate referrals to centers/experts specializing in the care of complex epilepsy disorders. Notably, early‐onset epilepsy and developmental delays that are characteristic of CDD may help to distinguish it from other DEEs in which seemingly normal development followed by regression is a typical feature.^5^ Other features that may distinguish CDD from those typical of other DEEs are shown in Table 3. As a result of the phenotypic variations between individuals with the same disease, it is important to emphasize the benefit of exact genotyping to confirm diagnosis, allowing care tailored to the disease and understanding progression of disease.

**TABLE 3:** Key clinical features of CDD, RTT, and Dravet syndrome.^3^, ^4^, ^78^

	CDD^3^	RTT^4^, ^78^	Dravet^3^, ^67^
Seizures	Tonic, epileptic spasms, generalized tonic–clonic, and/or focal seizures	Tonic–clonic, febrile, and focal seizures	Recurrent focal clonic febrile and afebrile seizures
EEG	Interictal: normal in early stage, progressively abnormal	EEG follows the four stages of disease (early onset, rapid destructive, stabilization, and late motor deterioration phases)	Normal at onset; interictal: focal, multifocal, and generalized; may appear after 2 years of age; may be triggered by photic stimulation
Age at onset	>3 months	6–12 months	1–20 months
Key clinical features	Very early onset of epilepsy; developmental delay, hypotonia, CVI, and hand stereotypies	Period of nearly normal development followed by regression with loss of acquired fine finger skill in early childhood, hand stereotypies, and characteristic intensive eye communication	Development and neurological examination are often normal at seizure onset; CVI; developmental slowing and consequent intellectual impairment; regression following episodes of status epilepticus; and gait disorder evolving to crouch gait, typically by late childhood to adolescence
Other essential testing	Pathogenic variant in *CDKL5* gene	Pathogenic variant in *MECP2* gene	Pathogenic variant in *SCN1A* gene (80–85%)

Abbreviations: CDD, CDKL5 deficiency disorder; CVI, cerebral (cortical) visual impairment; EEG, electroencephalogram; RTT, Rett syndrome.

Notably, when a CDD or another DEE is suspected, a center of excellence/specialist pediatric center or equivalent should be contacted for consultation/referral. Virtual consultations or telemedicine visits are a possibility where access to relevant specialist centers is limited. For example, referral information can be found through EpiCare (https://epi‐care.eu/ern‐epicare‐centres/). In addition, the Clinical Patient Management System is a teleconsultation platform for HCPs who are part of the EpiCARE ERNs, enabling remote care and virtual diagnosis via video consultations (https://metab.ern‐net.eu/clinical‐patient).

##### Use of genetic testing to confirm diagnosis

There are currently many different approaches to genetic testing that can be used to diagnose CDD. These include sequencing of the *CDKL5* gene, the use of a panel of known genes for epilepsy disorders or intellectual disability, whole exome or genome sequencing, or, in rare cases, chromosomal microarray.^47^

With over 100 genes implicated in DEEs,^47^, ^48^ one challenge in the use of genetic testing to confirm diagnosis is whether to perform broad‐spectrum screening or focus on specific genes.^49^ Another challenge is the varied availability of genetic testing between countries as many health authorities do not have access to or cannot afford genetic tests.

###### Expert opinion

Clinical and electroclinical features (Table 2) can be used to refine potential diagnoses, which aids the selection of genetic tests and suspected genes to investigate and confirm diagnosis. Genetic tests, when prescribed and interpreted by a specialist in the field, may be more cost‐effective than other clinical investigations and should be offered as soon as possible. If not done before, the test results should lead to a referral or consultation with a specialist center or equivalent for planning seizure management and multidisciplinary care.

Whole exome or genome sequencing may be considered a first‐tier clinical test given its improved diagnostic yield and reduced costs when used as the first approach for diagnosis of neurodevelopmental disorders.^50^, ^51^, ^52^ Chromosomal microarray, which detects copy number variations (microdeletions or duplications), may also be used in the diagnosis of unexplained epilepsy and complex phenotypes. However, chromosomal microarray may be redundant in certain settings as next‐generation sequencing approaches such as whole exome or genome sequencing enable single‐nucleotide variants and copy number variations to be detected in a single test.^52^

Support from relevant companies could help improve access to genetic testing. Local, tailored guidance on the type of genetic test that should be performed based on the patient's symptoms would also be beneficial.

There are some rare cases of people with CDD who do not present with seizures, but instead present with other neurodevelopmental symptoms of the disorder, such as intellectual disability or autism spectrum disorder. Therefore, it may also be relevant to perform diagnostic testing for CDD in cases that do not present with epilepsy onset.

##### Timing of diagnosis and genetic testing

Early diagnosis is crucial to support management of seizures, end the diagnostic odyssey for the families, and ensure disease‐specific counseling and coordination of multidisciplinary specialized care. Although there is currently no disease‐modifying treatment for CDD, a specific and accurate diagnosis is of interest for DEEs in general, in an age where precision medicine can dramatically improve the quality of life for patients and their families.

###### Expert opinion

A diagnosis should be made as early as possible, ideally during the first year of life and when seizures become refractory to treatment in all patients with suspected CDD and other similar early‐onset DEEs. In many European countries, all infants presenting with seizures and/or developmental delays should be offered a genetic test.

The etiology of up to 80% of patients with IESS can be diagnosed with genetic testing and neuroimaging, and such diagnoses can guide treatment.^53^

Educating pediatric neurologists in recognizing patients who should be referred to a center with expertise in rare genetic epilepsy syndromes is a key step to ensure a timely diagnosis and management. In addition, genetic testing should be ordered as early as possible to account for the time to obtain results from the testing laboratory.

##### Genetic testing in older individuals

Many older patients with CDD may have less access to or never received a genetic test to confirm etiological diagnosis,^54^ and, due to the varying degrees of severity and presence of variable features in the population, may still have a rather generic diagnosis of IESS or Lennox–Gastaut syndrome, or may have been registered as an unclassifiable DEE or a variant of Rett syndrome. A correct diagnosis in these individuals is still important to ensure disease‐specific management, particularly at a time when specific therapies are in development, and allowing comprehensive research of the population with CDD.

###### Expert opinion

When patients lack a confirmed diagnosis despite genetic tests being performed or the symptoms of the disease change, it is important to regularly repeat the clinical evaluations that were performed initially (such as genetic tests, imaging, video‐EEGs, and metabolic screenings) as molecular technology improves and, for example, methods that can detect previously unknown mutations/genetic variants become available.

##### Wider application of accurate diagnosis

Rare diseases are under‐reported and under‐resourced in healthcare information systems. It is, therefore, important to ensure standardized, detailed, and accurate reporting in healthcare systems that allows global comparison and sharing of data in a harmonized way. It facilitates the collection and storage of data for analysis and evidence‐based decision‐making. The International Classification of Disease (ICD) was created by the World Health Organization for monitoring the incidence and prevalence of diseases and related conditions, and storing diagnostic information for clinical, research, quality, and epidemiological purposes.^55^ In addition, the Orphanet nomenclature for rare diseases (ORPHAcodes), a European initiative, was established to contribute to the acceleration of clinical coding and research in rare diseases.^56^

###### Expert opinion

CDD coding has historically been inconsistent, leading to poor prevalence and mortality tracking, and difficulty accessing services for caregivers. Increased utilization and implementation of detailed codes for CDD specifically will improve appropriate access to services and treatments, and will improve research and future treatment opportunities by accurately tracking patient numbers. The ICD‐10 code for CDD is G40.42 and the ORPHAcode is ORPHA:505652.^57^

The innovative decision of the European Union to create the ERN EpiCARE opens new perspectives for early access of all patients to state‐of‐the‐art diagnostic tools and optimal therapeutic management.

#### 3.2.2 Management of seizures

##### Pharmacological seizure management

Prompt seizure management has shown evidence of beneficial effects on seizure onset and developmental delays in other DEEs,^58^, ^59^ but the link in CDD is poorly understood. Several general pharmacological treatments for managing seizures are available for patients with CDD and other DEEs. However, there are currently no approved drugs specifically for the treatment of seizures in patients with CDD in Europe.

###### Expert opinion

Owing to the inter‐individual variation in seizures, characterization of the seizure types and patterns, with video‐EEG, for example, is important to ensure optimal management of the disease. The treatment of seizures in CDD includes the general principles and approaches to treatment of epilepsy in a pediatric population. Common adverse effects of ASMs, although generally not severe,^15^ include increase in sedation,^60^ gastrointestinal upset,^60^ and seizure aggravation,^61^ and these require fine balancing with efficacy.

At the time of seizure presentation, ASMs are usually initiated in hospital, but given the highly refractory nature of seizures to ASMs in CDD, rapid and effective titration may be necessary. However, once the diagnosis is established and owing to short durations of hospitalization, titration may often be performed in home settings.

In addition to pharmacological treatment, non‐pharmacological approaches such as ketogenic diet and vagus nerve stimulation can be considered.^1^ Ketogenic diet is a good option at early stages of disease. Vagal nerve stimulation is most commonly reserved for older children (>4 years of age).

##### Multiple ASMs for seizure management

Epilepsy in CDD is highly refractory to ASMs, with less than half of all individuals experiencing a seizure‐free interval of at least 2 months.^62^ This refractory nature has an impact on the number and type of ASMs that are used. As reported in a US cohort study, the most frequently prescribed ASMs are broad spectrum, and the median number of ASMs prescribed over a lifetime is six.^14^ Polypharmacy is common practice, as observational studies of epilepsy/CDD treatment have shown that over 40% of patients are treated with multiple ASMs. It has been shown that polypharmacy is associated with a poorer quality of life than single therapy,^63^, ^64^ and careful consideration is needed around changes in ASM regimen.

###### Expert opinion

When changing and/or switching ASMs, frequency of seizures during screening period, adverse effects, drug–drug interactions, and impact on other outcomes (such as enhancing sleep disturbances, appetite, behavioral problems, or hypotonia) need to be considered,^65^ hence consultation of specialists with experience in managing seizures in CDD is crucial. Often, clinicians allow approximately 1 month of full dose before deciding to add a second drug. Balancing ASM efficacy with adverse effects is difficult, and in general, clinicians will not use more than three ASMs concomitantly in most patients. When using multiple ASMs as part of the treatment regimen, it can be beneficial to use ASMs that target different pathways to leverage additional effects.

When adding a new drug, slow titration is usually advisable to avoid adverse events. The influence of the drug at low doses must be observed, and if a drug does not work at low doses, its dose should not be up‐titrated as this will likely increase the risk of adverse effects. When side effects outweigh the benefits, the drug should be withdrawn. To avoid overlapping and antagonistic results between drugs, simultaneous initiation of more than one drug is not recommended.

###### Considerations for seizure management

The use of ASMs should be based not only on seizure types but also on comorbidities experienced by the patient and other concomitant medications used. The types of seizure in CDD often vary by age (Table 1). The most common seizure types occurring in patients with CDD are tonic seizures, epileptic spasms, and generalized tonic–clonic seizures.^41^

####### Expert opinion

Comorbidities and concomitant medication should be taken into consideration when deciding how to manage seizures. The use of concomitant medications will also vary by age, as the number of medications used in combination tends to increase with age. Behavioral problems or circadian/sleep disturbances are a major concern for patients with CDD and caregivers and should be taken into consideration. In addition, excessive sedation can increase seizures in some patients with features of Lennox–Gastaut syndrome.

In infants, epileptic spasms are often managed with steroids, followed by vigabatrin.^1^, ^36^, ^37^ Findings from a recent retrospective study of treatment with sodium‐channel blockers indicated favorable effectiveness and safety in a subgroup of patients with CDD‐related seizures (*n* = 6/19).^61^

Management of comorbidities should not be compromised because of prioritizing treatment of seizures. The route of administration of ASMs should be carefully considered with respect to comorbidities, for example, adolescents with gastrointestinal and swallowing issues may have nasogastric intubation or gastrostomy and would require a drug formulation compatible with this.

####### Reduction in seizure frequency in CDD

In people with CDD and other DEEs, success of ASMs can be very subjective, and effectiveness outside of seizure reduction is often based on developmental outcomes and reports from parents/caregivers on general well‐being. The impact of early or preventive treatment with ASMs in CDD has not yet been established and thus requires further research.

######## Expert opinion

Although regulatory agencies may use a numerical benchmark for reduction in the frequency of disabling seizures to define success, these are not always the outcomes most relevant to physicians and patients/caregivers. Outcomes such as reduction in certain types of seizures or achieving seizure‐free days may be more relevant to everyday life.

In addition, while seizure management is important from the perspective of the patient/caregiver, additional factors are equally crucial, including minimizing polypharmacy, improving quality of life, and improving associated behavioral/developmental issues such as sleep disturbances. Additionally, an improvement in nocturnal convulsive seizures (bilateral tonic–clonic seizures) is associated with a reduction in the risk of sudden unexpected death in epilepsy (SUDEP).

####### Setting expectations for seizure management

Seizure freedom is difficult to achieve and should not be the primary aim of CDD management in most patients. Clinicians' expectations regarding seizure management may differ from that of patients/caregivers, and these expectations may vary according to the age of the patient. From a patient/caregiver perspective, initial expectations regarding the potential of achieving seizure freedom after diagnosis are high but subside over time. To gain insights and form realistic expectations, patients and their families/caregivers should be encouraged to liaise with other families who have been through this journey before. A referral to clinical experts who can provide relevant information and counseling based on the diagnosis is key to supporting the family.

######## Expert opinion

Realistic expectations should be communicated to patients and caregivers at the start of treatment. A confirmed diagnosis is important to allow an accurate assessment of expectations related to seizure management and overall care.

#### 3.2.3 Multidisciplinary care

##### Multidisciplinary care—Children

In specialized pediatric centers, incorporation of multidisciplinary care has become commonplace; it involves a team of professionals working together to improve the quality of a child's life. However, time, expertise, resources, and constraints within healthcare systems and countries, as well as the variability of clinical symptoms and comorbidities, are key challenges to implementing quality multidisciplinary care. This is relevant to multidisciplinary care involved in any DEE.

###### Expert opinion

From experience, multidisciplinary care when coordinated by pediatric neurologists at specialist/tertiary centers works well, as they often have experience with this strategy and care needs. Patients not affiliated with specialist centers should have a community pediatrician (who is regularly liaising with a specialized center) that coordinates care between specialists. Furthermore, where multidisciplinary care is not readily available, families and professionals who care for patients must be willing to collaborate with each other.

A correct diagnosis is needed for an overall prognosis and an accurate care plan, treating the seizures, addressing and managing comorbidities, and supporting the family by addressing overall concerns.

The following aspects of care have previously been identified by international expert consensus as being crucial for multidisciplinary care in patients with CDD: neurorehabilitation, neuro‐ophthalmology, orthopedics, gastroenterology, physiotherapy, and occupational therapy.^36^ Psychological, neurobehavioral, cognitive, and developmental comorbidities in childhood epilepsies may also require additional multidisciplinary support. Aspiration pneumonia due to swallowing difficulties is one of the main causes of mortality in this population; therefore, pneumologists and gastroenterologists should be involved in the multidisciplinary team in some cases.

Furthermore, support based on social needs, such as transport, should also be considered.^36^ Ideally, to aid consistent care across Europe, a comprehensive action plan template for management of CDD should be developed, with flexibility to plan around individual clinical symptoms. There is a need to further research mortality rates and life expectancy of patients with CDD, and provide support and open sensitive discussion around palliative care with patients/caregivers.

##### Multidisciplinary care—Adults

The clinical features associated with CDD can change considerably from childhood to adulthood and the variability of the disease phenotype increases with age. Careful support and planning is required to successfully transition to adult care. Management of adult patients with CDD involves a greater focus on rehabilitation and behavioral therapy than in pediatric care.

###### Expert opinion

Regarding the evolution of CDD during adulthood, there is a need for specific adult phenotype studies and natural history. Notably, seizures remain highly refractory and there have been cases of new‐onset Parkinsonian features, as well as other respiratory, sleep, or cardiac disorders reported during teenage and adulthood CDD (manuscript in preparation).

Primary care physicians are not specialized to manage specific features of adult patients with CDD in the community, and given the rarity of this condition, a broad engagement and education of adult care clinicians is unrealistic. Therefore, clinicians who specialize in neurology or complex diseases in adults should be involved. For example, pharmacological and non‐pharmacological antiseizure treatment should be monitored in a specialist setting. Ideally, centers of excellence or equivalent should treat both pediatric and adult patients, or patients should be transitioned to adult neurologists with expertise in DEEs. When this is not possible, coordination between experts from different hospitals may aid efficient transition. Implementation of digitized systems for adult healthcare is crucial for transitioning and continuous care through adulthood (e.g., ensuring multidisciplinary teams have the information needed to provide holistic, personalized care).

Another challenge in transitioning to adulthood relates to adjustments in lifestyle. For example, adult patients with CDD usually frequent day centers or are placed in residential care. In these circumstances, school and even extracurricular activities that may provide distraction for children are no longer feasible. This can lead to the emergence of depression or other associated mental health problems.

##### Management of comorbidities

The focus of multidisciplinary care depends on the presence and severity of comorbidities, which will vary depending on the patient's age. Some comorbidities and clinical features seen in CDD also apply to other DEEs (e.g., digestive problems, CVI, sleep dysfunction, and behavioral dysregulation).^66^, ^67^, ^68^

###### Expert opinion

It is imperative that healthcare systems/providers gain an understanding of the needs of patients with CDD, and other DEEs, to allow implementation into the healthcare system. Psychiatrists or neuropsychologists should be part of the multidisciplinary team caring for people with CDD to support the behavioral aspects of the disorders, as these have a great impact on the quality of life for the individual with CDD as well as their families.

A previous international expert consensus reviewed the aspects of multidisciplinary care: for most comorbidities, it was agreed that a baseline assessment should be done with regular follow‐ups.^36^ These include assessments for gastrointestinal complications, feeding, and swallowing, with gastrointestinal and nutrition specialists involved at baseline. The follow‐up assessments should be done during regular clinic reviews. A detailed vision assessment should preferably be done by an ophthalmologist familiar with CVI. The choice of non‐verbal communication aids, such as gaze assistive technology, and the need for educational accommodations would be impacted by the severity of the CVI. The international expert consensus group agreed that sleep should be assessed at baseline and followed up annually. There is, however, no consensus currently on pharmaceutical treatments to improve sleep. Many of the experts also noted that neuropsychological and development assessments should be done at baseline and followed up regularly.^36^ The CDD Clinical Severity Assessment (CCSA) tool was developed through collaboration between HCPs and caregivers aiming to have a standardized assessment by clinician and caregiver of motor skills, cognition, behavior, vision, speech, and autonomic function; tools like this are intended for a standardized evaluation of therapeutic interventions on the various aspects of the disease.^69^

Families of people with CDD, who are not seeing an expert in the management of CDD/DEEs, find it challenging to receive the appropriate care. PAGs should be involved in understanding the needs of multidisciplinary care to support quality of life and help healthcare systems better understand the needs of the patient (and family) to prioritize management. Surveys among patients/caregivers would help further understand the unmet needs in multidisciplinary care.

## 4 DISCUSSION

The aim of this expert opinion piece was to map out the typical experience for patients with CDD across Europe, and to identify challenges and potential solutions/approaches to align the patient journey and standards of care.

Discussions on the established European patient journey raised some important issues. The journey related to presentation and diagnosis of CDD largely varies owing to factors such as variability of symptoms (including variable seizure types/patterns and severity of developmental disability) and lack of or delayed genetic testing. The individual differences in electroclinical features among people with CDD, in particular EEG activity, can also lead to differential diagnoses of encephalopathies caused by variants in other genes, such as *SCN2A* or *STXBP1*, in neonatal patients.^70^ This further emphasizes the need for early referral of all suspected DEEs to specialized centers and deep genetic testing to ensure correct identification of *CDKL5* variants in patients with CDD.

There are a handful of ASMs that have been approved for specific use in a range of DEEs, although no treatments are approved specifically for the management of seizures in CDD in Europe. Currently, for most genetic DEEs, targeted therapy means use of ASMs with known effectiveness for certain seizure types. In the United States, ganaxolone is the first ASM approved by the US Food and Drug Administration for the treatment of CDD‐related seizures in patients aged 2 years or over (given orally three times daily with dosage dependent on body weight).^71^ However, there is a future potential to expand treatment options and approaches based on a greater understanding of the etiology and genetic basis of individual DEEs.^72^, ^73^

Recommendations from an international panel of experts were reached by consensus on the management of patients with CDD, including the use of ASM. The panel found that the most favored ASMs were ganaxolone and cannabidiol^36^; neither are currently approved for the treatment of CDD in Europe. However, the lack of standardized treatment practices across Europe means that the management of seizures in CDD varies greatly between countries. Formal guidelines comprising definitions for successful treatment and treatment failure, as well as guidance on stopping/switching medication would help to standardize the management of seizures, similar to the consensus‐based guidelines for Rett syndrome,^74^ national guidelines for diagnosing and managing childhood epilepsies (including Dravet, Lennox–Gastaut, and IESS), and guidelines on establishing multidisciplinary care (as in TSC^75^).

Multidisciplinary care was defined as the third major element of the patient journey for people with CDD. Leveraging multidisciplinary care ensures a holistic view of the patient is considered, including all comorbidities. This approach is applicable no matter the age of the patient, although the focus of multidisciplinary care depends on the patients' age‐dependent clinical features.

The crucial aspects of multidisciplinary care in patients with CDD have been identified by expert consensus.^36^ However, access to multidisciplinary care is largely dependent on resources and/or funding, which varies from country to country. This is relevant to multidisciplinary care involved in any DEE or other developmental disorders. Establishing clinical networks, utilizing the potential of virtual clinical consultations with specialist clinics, and arranging virtual meetings for sharing of knowledge and data on evidence‐based management across Europe is necessary to ensure the timely diagnosis of people with CDD and appropriate management of all aspects of disease.

More research into life expectancy and morbidities associated with CDD is also needed, as patients with CDD are at high risk of SUDEP owing to high seizure frequency and severity, although this risk seems lower than with other DEEs.^1^, ^36^ The annual risk of SUDEP among patients with CDD remains unknown owing to paucity of data and reasons for sudden infant death are often unclear.

While the outlined journey reflects current experience, it is not necessarily the ideal patient journey; there is a need to align widely on treatment goals. Owing to the disparity across Europe and within countries, communication from the European Commission has recently been sent to health ministers petitioning for collaboration and development of national networks; local, national, and international action is required. EpiCARE is building national care pathways in 26 countries across Europe with the mission to help patients with rare or low‐prevalence complex diseases (www.epi‐care.eu). More support is needed from relevant parties (such as HCPs, PAGs, pharmaceutical companies, and scientific researchers) to implement these national care pathways as part of the optimized European patient journey. Notably, patients with other DEEs experience similar symptoms and require access to similar care. Therefore, some, but not all, elements of the journey will be applicable to other DEEs.

Support from health authorities is crucial for implementing guidelines, thus, health authorities are encouraged to collaborate across different countries within Europe and internationally to achieve this. A European patient registry has been established by EpiCARE and will be useful for generating data and evidence to support development of standardized guidelines. Systematic and harmonized data collection is supported by other European health data initiatives aiming at improving patient records, with incorporation of national registries into larger international registries. While the focus of the patient journey reported herein has been within Europe only, it is important to note that different practices and standards are likely to occur outside of Europe. Additionally, although many of the themes discussed refer to standardizing clinical practice (e.g., use of ASM), it should be acknowledged that a personalized approach should be taken, considering the comorbidities specific to each individual patient.

## 5 CONCLUSIONS

With a lack of evidence to develop standardized guidance on quality care for people with CDD within the European setting, a group of experts in the field convened to propose recommendations that may help to address this unmet need. Initial opinions and experiences derived from clinician and patient/caregiver perspectives were used to map the optimized patient journey for people with CDD in Europe, focusing on three main elements: presentation/diagnosis, seizure control, and multidisciplinary care. Further discussion between the experts highlighted numerous disparities in management approaches and resources across different European countries. The panel recognized that while early confirmation of the electroclinical diagnosis through genetic testing is especially beneficial to optimizing patient outcomes, the availability of genetic testing varies widely across countries. Likewise, there was consensus that multidisciplinary care should take on a personalized approach based on patients' comorbidities, but time and resources often limit the care that is available. To achieve standardization of CDD management according to the optimized patient journey, evidence generation is required for European guidelines that align on realistic treatment goals, diagnostic criteria, and management approaches, and can be adapted for different settings.

## AUTHOR CONTRIBUTIONS

All authors listed have made a substantial, direct, and intellectual contribution to the work and approved it for publication.

## FUNDING INFORMATION

The editorial assistance for this manuscript was provided by AXON Communications, supported by Orion Corporation Orion Pharma.

## CONFLICT OF INTEREST STATEMENT

AAS has received financial support for educational and research activities from Angelini Pharma, Blueprint Genetics, Health in Code, Jazz Pharmaceuticals, Orion Pharma, PTC Therapeutics, and UCB Pharma; GJK has received honoraria for advisory boards from Desitin Arzneimittel, GW Pharmaceuticals, and Orion Pharma, support of research activities from Nutricia and Eisai, and support of seminars from Desitin Arzneimittel, GW Pharmaceuticals, and Zogenix; NS has served on scientific advisory boards for GW Pharmaceuticals, BioMarin, Arvelle, Marinus, Orion Pharma, and Takeda, received speaker honoraria from Eisai, BioMarin, Livanova, and Sanofi, and served as an investigator for Zogenix, Marinus, BioMarin, UCB, and F. Hoffmann‐La Roche. RK has received speaker honoraria from Eisai, Jazz Pharmaceuticals, Orion Pharma, and UCB, and honoraria for advisory boards/consultation from Angelini Pharma, Eisai, Jazz Pharmaceuticals, Orion Pharma, and UCB; RN has received lectures fees from Advicenne, Eisai, GW Pharmaceuticals, Lundbeck, Nutricia, UCB, and Zogenix, consulting fees from Biogen, Biocodex, Lundbeck, Novartis, Orion Pharma, Supernus, Takeda, and UCB, received unrestricted educational grants from Eisai, GW Pharmaceuticals, Livanova, Orion Pharma, Servier, and UCB, served as principal investigator on clinical trials for Biocodex, Eisai, GW Research Ltd, UCB, and Zogenix, and received grants from EU FP7 program, Horizons 2020 Program, European joint program on rare diseases program (EJP‐RD), Imagine Institute Program and Bettencourt Schueller Foundation, and FAMA Philantropic fund for Epilepsy Chair at Imagine. RSM has received consulting fees from Orion Pharma and UCB, and speaker honoraria from UCB, Orion Pharma, Eisai, and Angelini Pharma; SJ has received honoraria for advisory boards from Jazz Pharmaceuticals; SA has received funding from BioMarin, Boston Scientific, CDKL5 UK, Desitin, GW Pharmaceuticals, Ipsen, LivaNova, Medtronic, National Institute for Health Research, Novartis, Nutricia, Orion Pharma, PTC Therapeutics, TSA, and UCB; SSB has received personal fees from Eisai, Desitin Arzneimittel, GW/Jazz Pharmaceuticals, UCB, Marinus, Takeda, and Zogenix. AA, CAP, PB, and SL reported no conflicts of interest.

## ETHICS STATEMENT

We confirm that we have read the Journal's position on issues involved in ethical publication and affirm that this report is consistent with those guidelines.
